# Seasonal community structure and environmental associations of putative pathogenic microorganisms in the Shelf-Margin Sea of the Northwestern South China Sea

**DOI:** 10.3389/fmicb.2026.1980205

**Published:** 2026-10-26

**Authors:** Xiaojie Deng, Qingxiang Chen, Jannatul Ferdoush, Chongqiu Huang, Yanfeng Tang, Guozhi Zhang, Yan Zhu, Gonglingxia Jiang, Ke Dong, Nan Li, Laizhen Huang

**Affiliations:** 1 Key Laboratory of Climate, Resources and Environment in Continental Shelf Sea and Deep Sea of Department of Education of Guangdong Province, Department of Oceanography, Key Laboratory for Coastal Ocean Variation and Disaster Prediction, College of Ocean and Meteorology, Guangdong Ocean University, Zhanjiang, China; 2 Department of Biological Sciences, Kyonggi University, Suwon-si, Republic of Korea

**Keywords:** complexity, co-occurrence network, marine putative pathogenic microorganisms, PLS-PM, stability

## Abstract

Marine pathogenic microorganisms are crucial to ecological health and fishery aquaculture. However, the composition and influencing drivers for pathogenic microorganisms in the Shelf-Margin Sea of the Northwestern South China Sea (SMS-NWSCS) are still unclear. This study applied 16S rRNA and ITS gene high-throughput sequencing combined with multiple bacterial pathogen detection (MBPD) and FUNGuild database to explore putative pathogenic microorganisms in the SMS-NWSCS and to investigate the main environmental associations of the composition, diversity, complexity and stability of putative pathogenic microbial communities. Results showed that *Escherichia coli* (31.77%), *Pseudomonas fluorescens* (16.25%) and *Sphingomonas paucimobilis* (6.90%) were dominant putative pathogenic bacteria, while *Pseudozyma sp.* (48.36%) and *Malassezia globosa* (48.34%) dominated the putative pathogenic fungal communities. The putative pathogenic bacterial community exhibited higher alpha diversity in winter, while the fungal community showed greater alpha diversity in summer. Beta diversity analysis revealed significant seasonal dynamics in both putative pathogenic bacterial and fungal communities (bacteria: R^2^ = 0.12, *p* = 0.001; fungi: R^2^ = 0.08, *p* = 0.007). Spearman’s correlation analysis suggested that a suite of environmental factors, including temperature, pH, DO, *Chla* and nutrients, played significant roles in influencing putative pathogenic microbial community structure. PLS-PM analysis demonstrated that bacterial alpha diversity was positively associated with community complexity (R^2^ = 0.10, *p* < 0.01) and stability (R^2^ = 0.45, *p* < 0.001), and both alpha and beta diversity (R^2^ = 0.10, *p* < 0.05; R^2^ = 0.21, *p* < 0.001) showed positive correlations with the stability of the putative pathogenic fungal community. Our study reveals the seasonal patterns of putative pathogenic microorganisms in the SMS-NWSCS and enhances the understanding of their community maintenance mechanisms.

## 1 Introduction

Marine pathogenic microorganisms, mainly including pathogenic bacteria and fungi, are a vital component of marine ecosystems and pose a substantial threat to global public health and fishery aquaculture (Zgouridou et al., 2022). Currently, research on marine pathogenic microorganisms mostly focuses on the monitoring and identification of individual pathogenic microorganisms and the investigation of the virulence regulation mechanism (De Silva et al., 2022). For example, Morrison et al. (2024) examined the relationships among hydrographic, meteorological, and biological correlates of *V. vulnificus* and *Vibrio parahaemolyticus* in the Eastern Mississippi Sound System. Pernice et al. (2024) studied the potential pathogenic fungus *Gjaerumia minor* in the bathypelagic waters of the Equatorial Pacific, focusing on the morphological characteristics and distribution patterns. Exploring marine microbial diversity is critical for maintaining the stability of marine ecological services and sustaining ecosystem functions (Fan et al., 2022). However, the diversity and maintenance mechanisms of the marine pathogenic microbial community are remain largely unknown.

Previous molecular ecological studies of marine pathogenic microbial communities primarily focused on a certain group using specific primers. For example, Qin et al. (2024) demonstrated that nutrients had a crucial effect on the diversity and community stability of the marine Vibrio. Recently, the widespread use of the multiple bacterial pathogen detection (MBPD) database and the FUNGuild for pathogenic microorganism identification has led researchers to comprehensively study the marine pathogenic microbial community (Nguyen et al., 2016; Yang et al., 2023). For instance, employing MBPD methodology, Zhao et al. (2024) identified that the ASVs accounting for 41.22% of the total reads in six sludge samples were classified as pathogenic bacteria. Guo Y. et al. (2022) revealed that pathogenic bacterial communities in the Bohai Sea exhibit extreme temporal heterogeneity and are regulated by environmental factors. However, systematic insight into ecological resilience of marine pathogenic microbial communities and their main influencing factors in the SMS-NWSCS remains limited. The stability and complexity of the co-occurrence network are key indicators for measuring the ability of microbial communities to resist environmental disturbances and anthropogenic impacts (Jiang et al., 2024). Community stability is characterized by robustness, while total cohesion is considered an indicator of complexity (Herren and McMahon, 2017). Numerous studies have shown that community stability and complexity are affected by a variety of environmental factors (Oliver et al., 2015). For instance, Liu et al. (2023) found that the large salinity gradient exerted a significant influence on the topological properties and stability of the microeukaryotic co-occurrence network. Wang et al. (2024) reported that prokaryotic microbiomes exhibited greater molecular network stability and complexity relative to microeukaryotes, driven by temperature gradients in the Bohai Sea and Yellow Sea. However, the stability and complexity of the marine pathogenic microbial community structure in the SMS-NWSCS are still unclear.

The SMS-NWSCS, located in the western part of Guangdong Province, is a subtropical shelf sea characterized by rich biodiversity and complex ecosystems. The region is significantly coupled by the East Asian monsoon, the coastal current and the South China Sea warm current, with frequent land-sea material exchange, complex hydrodynamic conditions and Pearl River freshwater input, making it a critically important fishery resource area (Zhang et al., 2026). Therefore this study applied 16S rRNA and ITS gene high-throughput sequencing combined with MBPD and FUNGuild databases to analyze the marine putative pathogenic microbial community in surface seawater from the SMS-NWSCS in 2023, and aims to (i) identify the species composition and diversity of marine putative pathogenic microorganisms in the SMS-NWSCS, (ii) reveal the network topological features of the marine putative pathogenic microbial community, and (iii) explore the main environmental associations of diversity, stability and complexity of the marine putative pathogenic microbial community. Overall, this study provides new insights into the mechanisms that maintain the diversity and ecological resilience of putative pathogenic microorganisms in marine environments and offer reference data to support subsequent pathogen monitoring and risk assessment.

## 2 Materials and methods

### 2.1 Sampling sites and environmental parameters

Surface seawater (3 m) was sampled from 7 sites in the SMS-NWSCS (Figure 1), with 42 samples collected in both summer and winter. For each site, three replicate samples were collected utilizing a SBE 32 Carousel Water Sampler. All 42 seawater samples were used for 16S rRNA and ITS sequencing. Prefilter each seawater sample (1 L) through 3-μm filters (Port Washington, NY, United States) to remove debris and larger organisms. Take 500 mL of the prefiltered water sample to filter it through a 0.22-μm filter membrane (Millipore Corporation, Billerica, MA, USA), and store the membrane at −80°C for later use. A portable meter (556 MPS; YSI, USA) was used to determine environmental parameters such as temperature (°C), salinity, dissolved oxygen (DO, mg/L) and pH. The levels of phosphate (PO_4_^3–^-P, μmol/L), nitrate (NO_3_^–^-N, μmol/L) and ammonium (NH_4_^+^-N, μmol/L) were measured by a flow injection analyzer (SealAA3, Germany). Chlorophyll a (Chla, μg/L) was analyzed using a spectrophotometric method.

**FIGURE 1:**
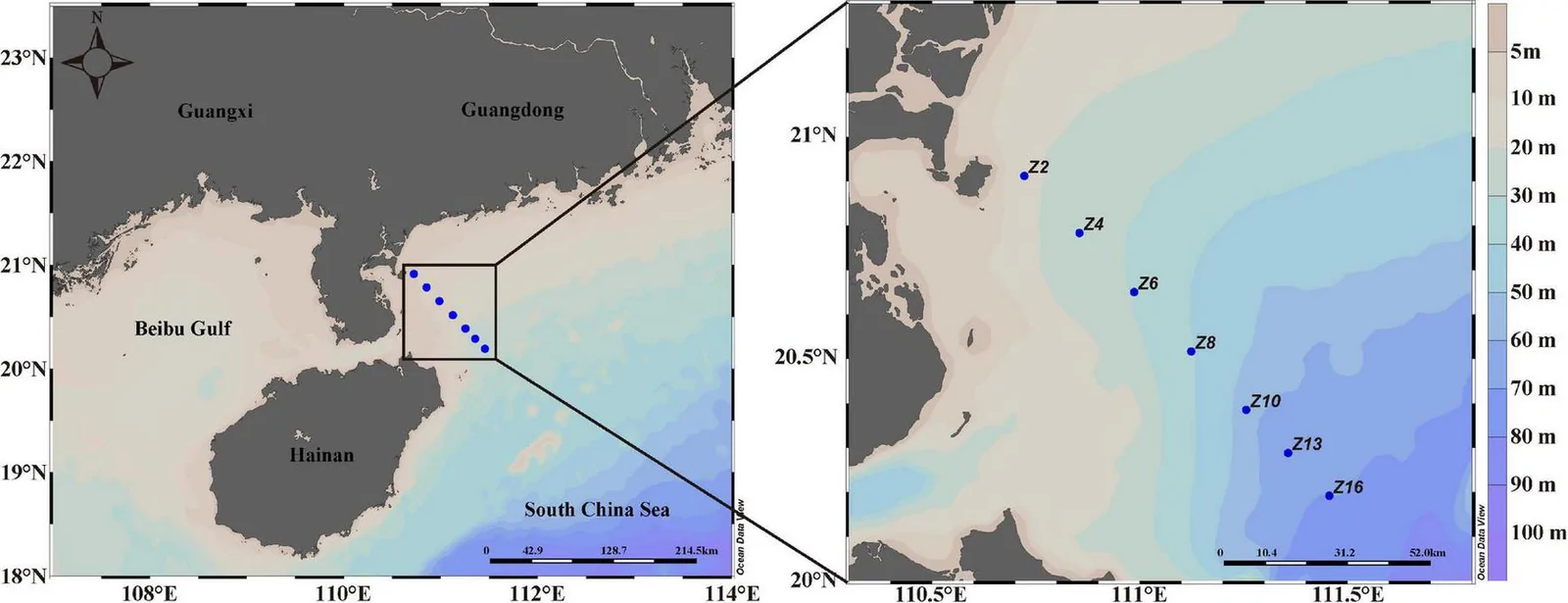
Localization of the 7 sites in the SMS-NWSCS.

### 2.2 DNA extraction and PCR amplification

Environmental DNA was extracted from samples through a DNeasy PowerWater Kit (QIAGEN, United States) with 0.22-μm polycarbonate membranes. NanoDrop Spectrophotometer (Thermo Fisher Scientific) was used for DNA concentration and purity. The primer 16S-F (5′-AGAGTTTGATCMTGGCTCAG-3′) and 16S-R (5′-TACGGYTACCTTGTTACGACTT-3′) were used to amplify the V3-V4 regions of the 16S rRNA gene. The PCR reaction mixture (total volume, 20 μL) contained 2 μL of template DNA, 6 μL of ddH2O, 10 μL of 2 × Taq PCR Mastermix (TianGen, China), and 2 μL of each primer. The PCR conditions included 1 min initial activation at 94°C, 35 cycles of denaturation for 30 s at 95°C, annealing for 30 s at 56°C, 30 s extension at 72°C, and a final extension for 10 min at 72°C. Primers ITS1F (5′-CTGGTCATTTAGAGAAGTAA-3′) and ITS2 (5′-GTGCGTTCTTCATCGATGC-3′) were used for the amplification of the ITS1 region of fungal DNA. The thermal cycling protocol consisted of an initial denaturation at 95°C for 5 min, followed by 30 cycles of 94°C for 30 s, 50°C for 45 s, 72°C for 2 min, with a final extension at 72°C for 10 min. Ultrapure water served as the negative control to eradicate possibility of false-positive results. The reaction products were confirmed with 2% agarose gel electrophoresis and visualized using a UV light and gel imaging system.

### 2.3 High-throughput sequencing

The purified library was prepared according to the manufacturer’s instructions and sequenced on an Illumina MiSeq platform at Majorbio Biotech (Shanghai, China). Raw sequence data were processed using QIIME 2 (version 2024.2) with standardized quality control pipelines for bacterial 16S rRNA and fungal ITS datasets separately. For the 16S rRNA, sequences with primer mismatches or lengths shorter than 300 bp were removed. For the fungal ITS, sequences shorter than 200 bp were discarded to filter incomplete and low-quality amplicons. For sequencing statistics, a total of 2,569,143 raw 16S rRNA reads were generated, and 2,568,009 high-quality reads were retained after quality filtering, with an average sequencing depth of 61,143 reads per sample. For the ITS, a total of 3,339,621 raw reads were obtained, and 3,338,982 high-quality reads remained after filtering, with an average sequencing depth of 85,615 reads per sample. After further dereplication and chimera filtering in QIIME2, the reads were clustered into OTUs with a 97% similarity.

Taxonomic assignment of bacterial OTUs was completed against the Silva 16S database (v138.1), while the fungal OTUs were assigned against the Unite database (v.9.0). All sequence data were deposited under BioProject Accession numbers PRJNA1469362 and PRJNA1469374 in GenBank.

### 2.4 Statistical analysis

Shannon and Richness indices of marine putative pathogenic microbial communities were calculated by R software (version 4.3.2) and were selected to represent alpha diversity in this study. To show the connection between environmental factors and alpha diversity, Spearman heatmap and simple linear regression were performed using “pheatmap” and “ggplot2.” The Benjamini–Hochberg false discovery rate (FDR) correction was applied to control for multiple comparisons. Comparisons between different communities were conducted by Bray–Curtis distance, Principal Coordinate Analysis (PCoA) and Permutational Multivariate Analysis of Variance (PERMANOVA), and the results were visualized by “ggplot2.” Particularly homogeneity of multivariate dispersion was tested to confirm the assumptions of PERMANOVA.

The relationship between marine putative pathogenic microbial community structure and environmental factors was analyzed by mantel test. Co-occurrence network analysis was performed separately for summer and winter putative pathogenic bacterial and fungal communities with the “Hmisc” and “igraph” packages. Before network construction, low-frequency taxa were filtered: only taxa detected in no fewer than 10 samples were retained for subsequent correlation analysis. Pairwise associations were calculated using Spearman’s rank correlation. Correlations were retained only when the absolute correlation coefficient (|r| < 0.7 and *P* < 0.01). Multiple testing correction was applied in this analysis. Positive and negative correlations were distinguished and preserved separately in the network. Identical filtering criteria and correlation thresholds were used for bacterial and fungal networks. The network graph was visualized using Gephi 0.9.2 software. Total cohesion was used to determine the complexity and the robustness of the community was calculated by using “igraph” to indicate its stability. Partial least squares path modeling (PLS-PM) was performed using the “plspm” package to evaluate the direct and indirect effects of environmental factors, microbial diversity, and community complexity and stability. Identical inner-model structures were fitted separately for putative pathogenic bacterial (*n* = 861) and fungal (*n* = 741) communities. Each model contained six latent variables: Water Properties, Nutrients, Alpha diversity, Beta diversity, Complexity, Stability. The Water Properties included temperature, salinity, pH, DO, and *Chla*, representing basic seawater physicochemical conditions. The Nutrients comprised nitrate, ammonium, and phosphate. Multicollinearity was checked by variance inflation factor (VIF). Bootstrap resampling with 1000 replicates was performed to assess the significance of path coefficients. Model fit was assessed by R^2^ of the latent variables and the goodness-of-fit (GOF).

### 2.5 Putative pathogenic microorganisms analyses

Putative pathogenic microorganisms were screened using MBPD (v.1.0) and FUNGuild (v.1.1) databases. For bacteria, the MBPD database was used to identify putative pathogenic taxa from 16S taxonomic annotations. A similarity cutoff of 0.9 was used for taxonomic matching. Taxa with records of pathogenicity in MBPD were retained, while ambiguous and unresolved species-level assignments were excluded. For fungi, FUNGuild was applied to ITS taxonomic profiles. Consistent with standard FUNGuild protocols, only functional assignments with “Highly Probable” and “Probale” confidence ranks were retained, and taxa labelled as “Possible” were removed to minimize false positives. Taxa without guild information or with uncertain classification were discarded. The retrieved taxa were defined as putative pathogenic microorganisms for subsequent analyses.

## 3 Results

### 3.1 Composition and diversity of marine putative pathogenic microorganisms in the SMS-NWSCS

81 samples from two seasons (summer and winter) were obtained from the SMS-NWSCS. After quality filtering and clustering at a 97% sequence similarity threshold, these bacterial sequences were classified into 12,949 OTUs and fungal sequences were classified into 3,048 OTUs. The OTUs accounting for 46.68% were classified as putative pathogenic bacteria, while about 6.20% were classified as putative pathogenic fungi. Good’s coverage values were 98.29 ± 1.39 and 99.45 ± 0.49, indicating that the vast majority of the putative pathogenic bacterial and fungal communities were recovered. At the species level, the top 10 putative pathogenic bacterial taxa were identified as the dominant components of the bacterial communities: *Escherichia coli*, *Pseudomonas fluorescens*, *Sphingomonas paucimobilis*, *Roseobacter denitrificans*, *Paracoccus sanguinis*, *uncultured Candidatus Pelagibacter sp.*, *Clavibacter michiganensis*, *Psychrobacter phenylpyruvicus*, *Bacillus pumilus* and *Acinetobacter venetianus* (Supplementary Figure 1). *E. coli* was the most abundant species between summer (38.67 %) and winter (26.34 %), and decreased from summer to winter. *P. fluorescens* increased from summer (15.13 %) to winter (17.14 %). While *S. paucimobilis* decreased from summer (7.34 %) to winter (6.56 %) (Figures 2A–C). For the putative pathogenic fungal community, the top 10 putative pathogenic fungal species were identified as the most abundant components across samples: *Pseudozyma sp.*, *Malassezia globosa*, *Cucurbitaria piceae*, *Engyodontium album*, *Malassezia restricta*, *Ceratobasidiaceae sp.*, *Malassezia sp.*, *Letendraea cordylinicola*, *Hermetothecium mikaniae micranthae*, and *Moesziomyces parantarcticus* (Supplementary Figure 1). *Pseudozyma sp.* and *C. piceae* both decreased from summer (50.22%, 1.87%) to winter (45.25%, 0.23%), while *M. globosa* increased from summer (47.42%) to winter (49.88%) (Figures 2D–F).

**FIGURE 2:**
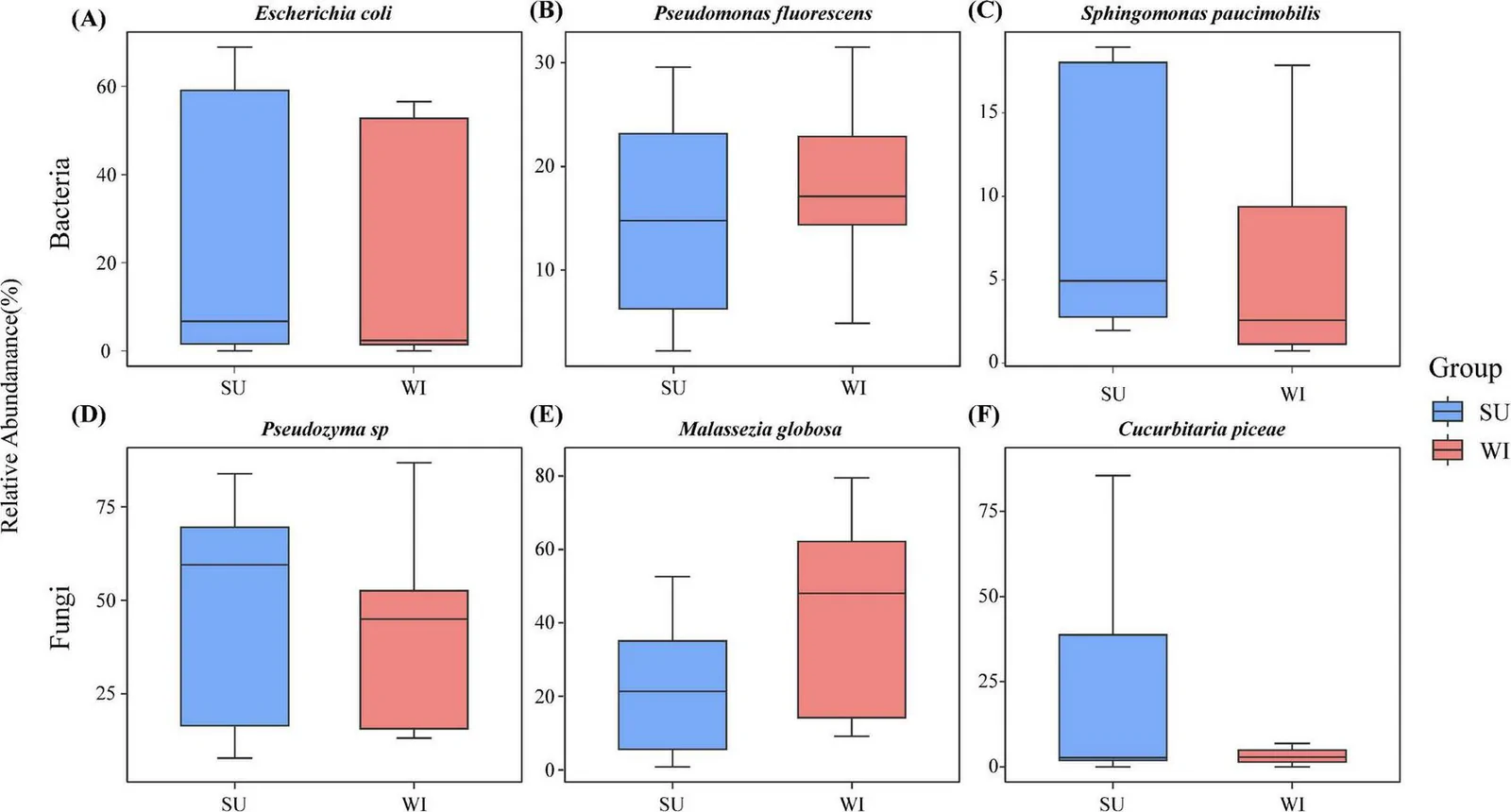
**(A–C)** Relative abundances of the 3 most abundant marine pathogenic bacterial species in different seasons. **(D–F)** Relative abundances of the 3 most abundant marine pathogenic fungal species in different seasons. SU, summer; WI, winter.

Shannon and Richness were calculated for assessing the alpha diversity of marine putative pathogenic microorganisms. Comparison of the median values from boxplots showed that the Shannon and Richness indices of the putative pathogenic bacterial community were numerically higher in winter than in summer. For putative pathogenic fungi, median Shannon and richness values were numerically higher in summer than in winter. Statistically significant seasonal differences were only detected for fungal richness; no significant seasonal differences were found for the remaining alpha diversity indices (Figure 3). PCoA of beta diversity (Bray-Curtis distance) showed differences in marine putative pathogenic microbial community structures between summer and winter. The PCo1 and PCo2, first two principal coordinates, explained 10.90% and 10.64% of the total variance. The PERMANOVA (R^2^ = 0.12, *p* = 0.001) tests also revealed significant differences in putative pathogenic bacterial community structure between the Summer and Winter (Figure 4A). In the marine putative pathogenic fungal community, the PCo1 and PCo2, first two principal coordinates, explained 12.47% and 12.24% of the total variance, while PERMANOVA (R^2^ = 0.08, *p* = 0.007) tests exhibited significant differences in putative pathogenic fungal community structure between the Summer and Winter (Figure 4B).

**FIGURE 3:**
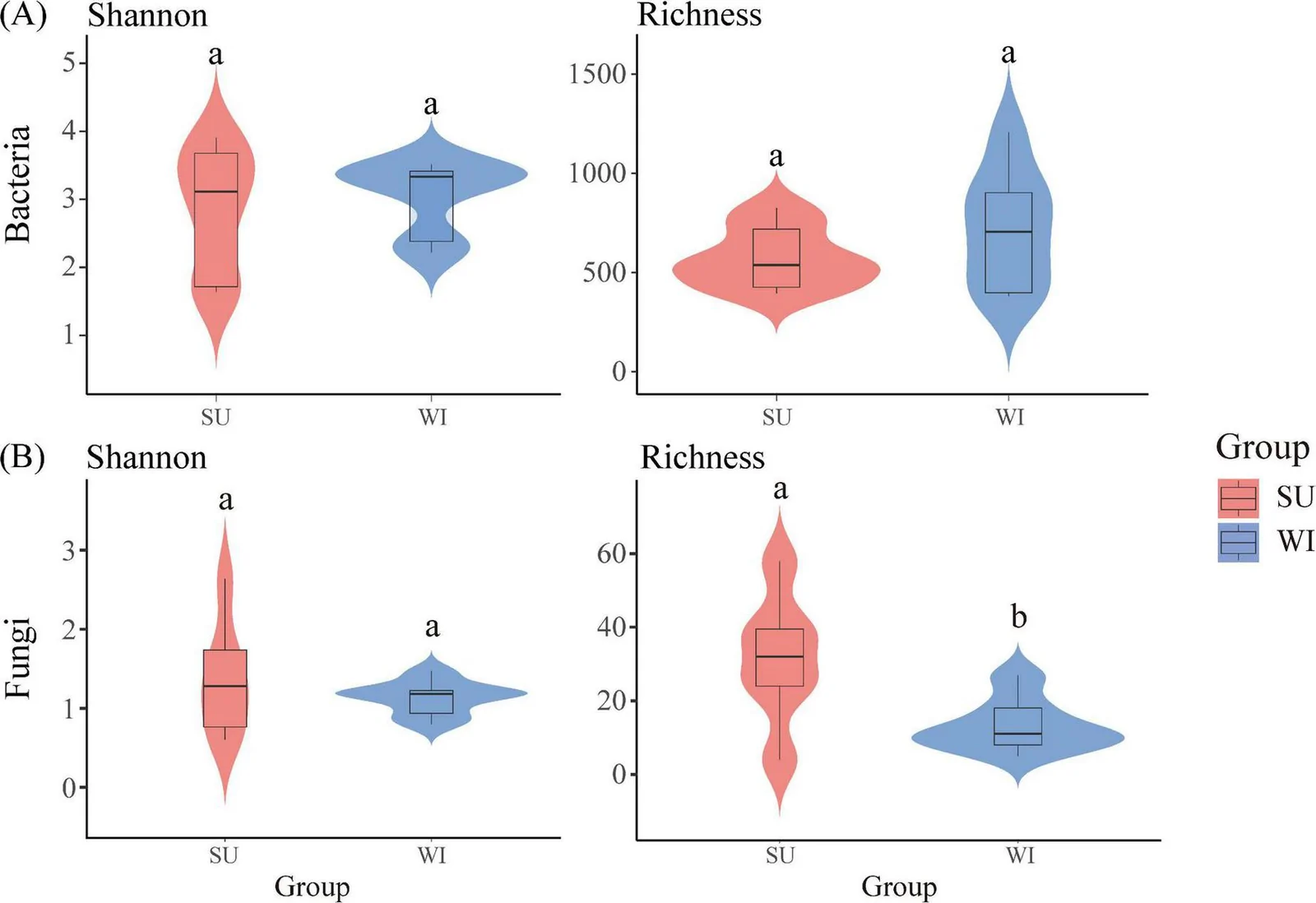
The alpha diversity (Shannon and Richness) of the marine pathogenic microbial communities across seasons. **(A)** Marine pathogenic bacterial community. **(B)** Marine pathogenic fungal community. The alpha diversity is shown by a violin boxplot. Groups sharing the same lowercase letter are not significantly different (*p* > 0.05), whereas different letters represent significant differences (*p* < 0.05). SU, summer; WI, winter.

**FIGURE 4:**
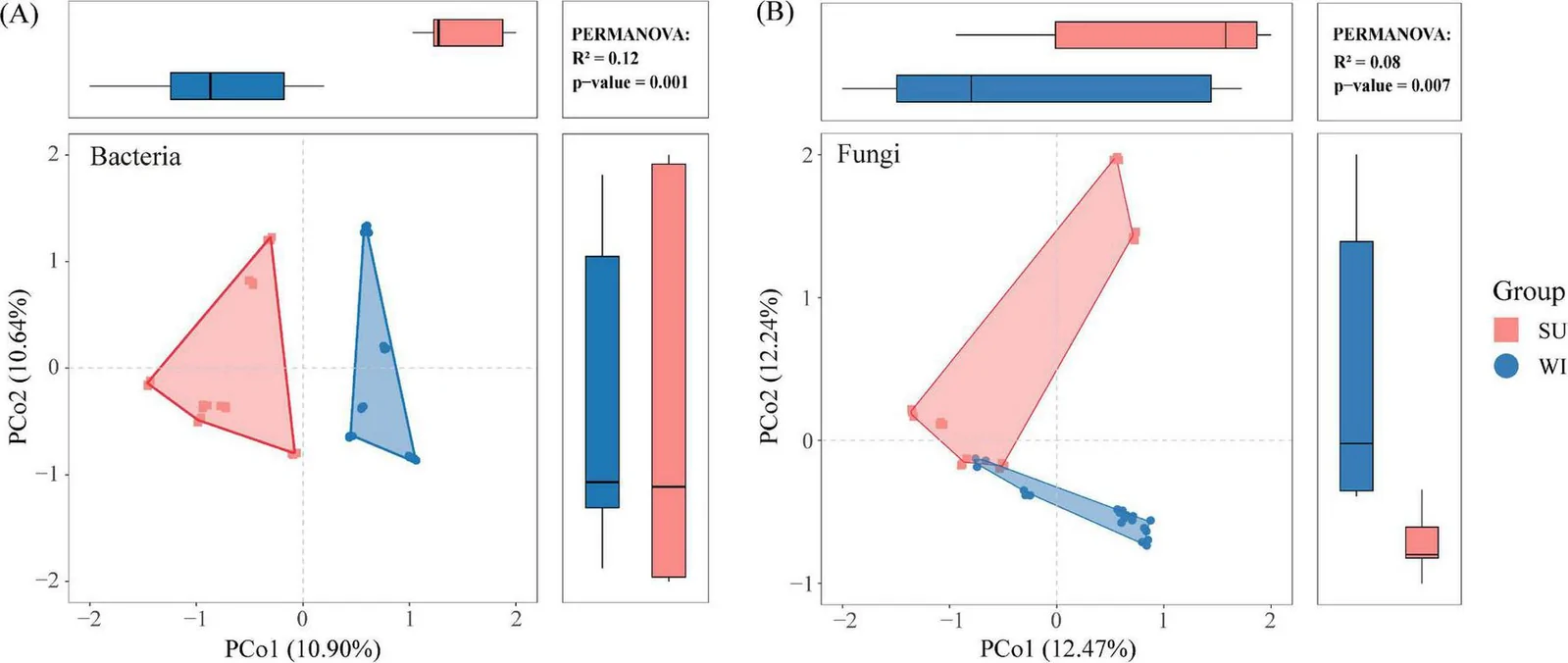
Differences in marine pathogenic microbial community structures between summer and winter (PCoA). **(A)** Marine pathogenic bacterial community. **(B)** Marine pathogenic fungal community.

### 3.2 Network topological features of the marine putative pathogenic microbial community in the SMS-NWSCS

The co-occurrence networks were established for each group (summer and winter) among marine putative pathogenic microbial OTUs (Figures 5A, B, 6A, B). In the putative pathogenic bacteria, the winter network had a greater average degree (14.99) while the average degree of summer was 10.68. The summer network showed the same number of modules (5) as winter but higher modularity indices (0.555) than winter (0.456) (Supplementary Table 3). For the putative pathogenic fungal networks, the summer network exhibited a greater average degree (34.38), whereas the average degree of winter was 14.40. However, the density of the winter (0.244) was higher than that of the summer network (0.237). The summer network contained 5 modules, fewer than the winter network (6 modules). In contrast, the modularity indices of the summer network (0.614) were higher than those of the winter network (0.496) (Supplementary Table 3).

**FIGURE 5:**
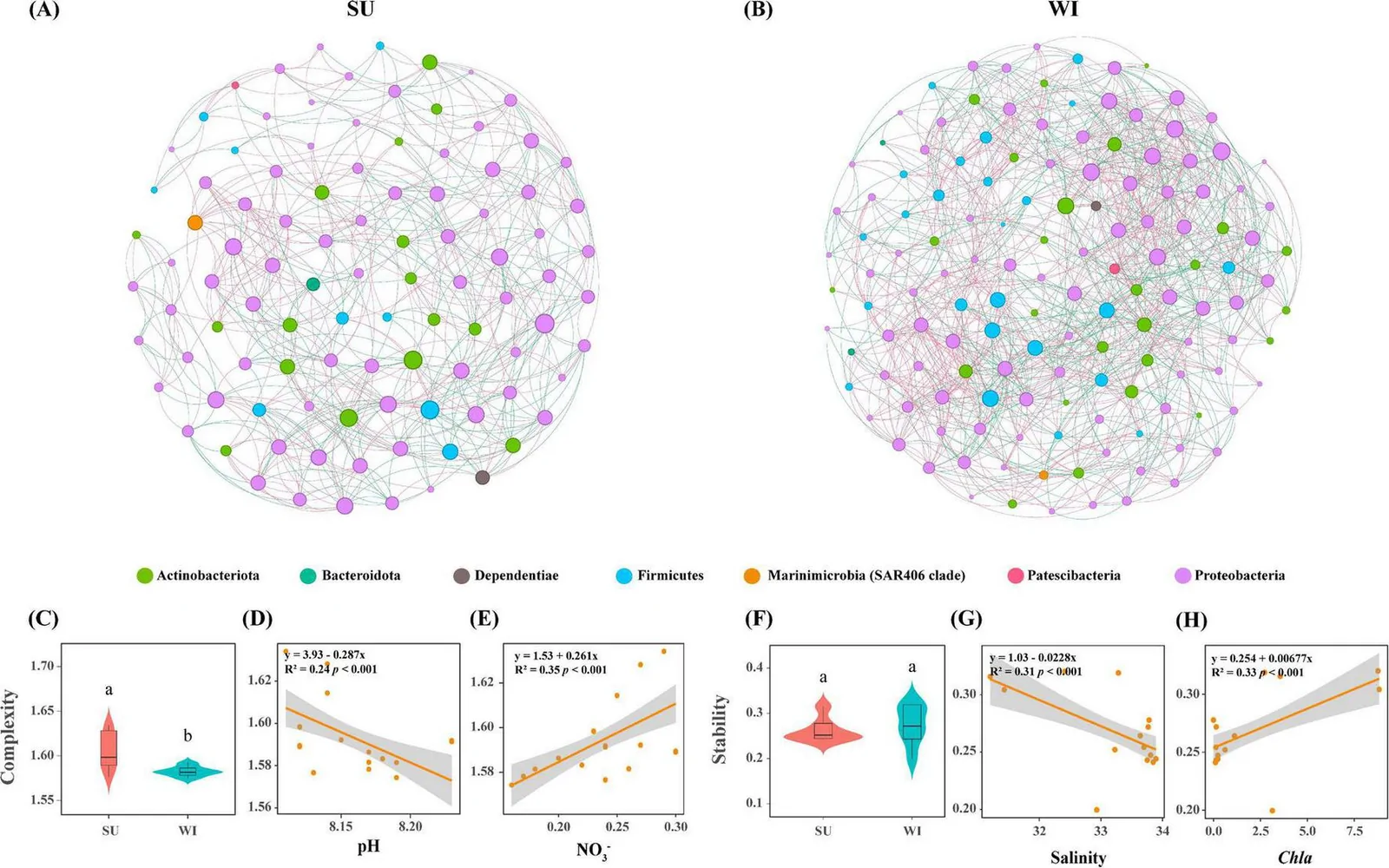
**(A,B)** Co-occurrence network of summer and winter groups of the marine pathogenic bacterial. The colors of node represent species belonging to the same module within each network. The pink and green lines represent positive and negative correlations, respectively. **(C)** Complexity in different seasons is presented by a box plot. **(D,E)** Linear regression analyses of complexity against pH and nitrate. **(F)** Stability in different seasons is presented by a box plot. **(G,H)** Linear regression analyses of stability against salinity and *Chla*.

**FIGURE 6:**
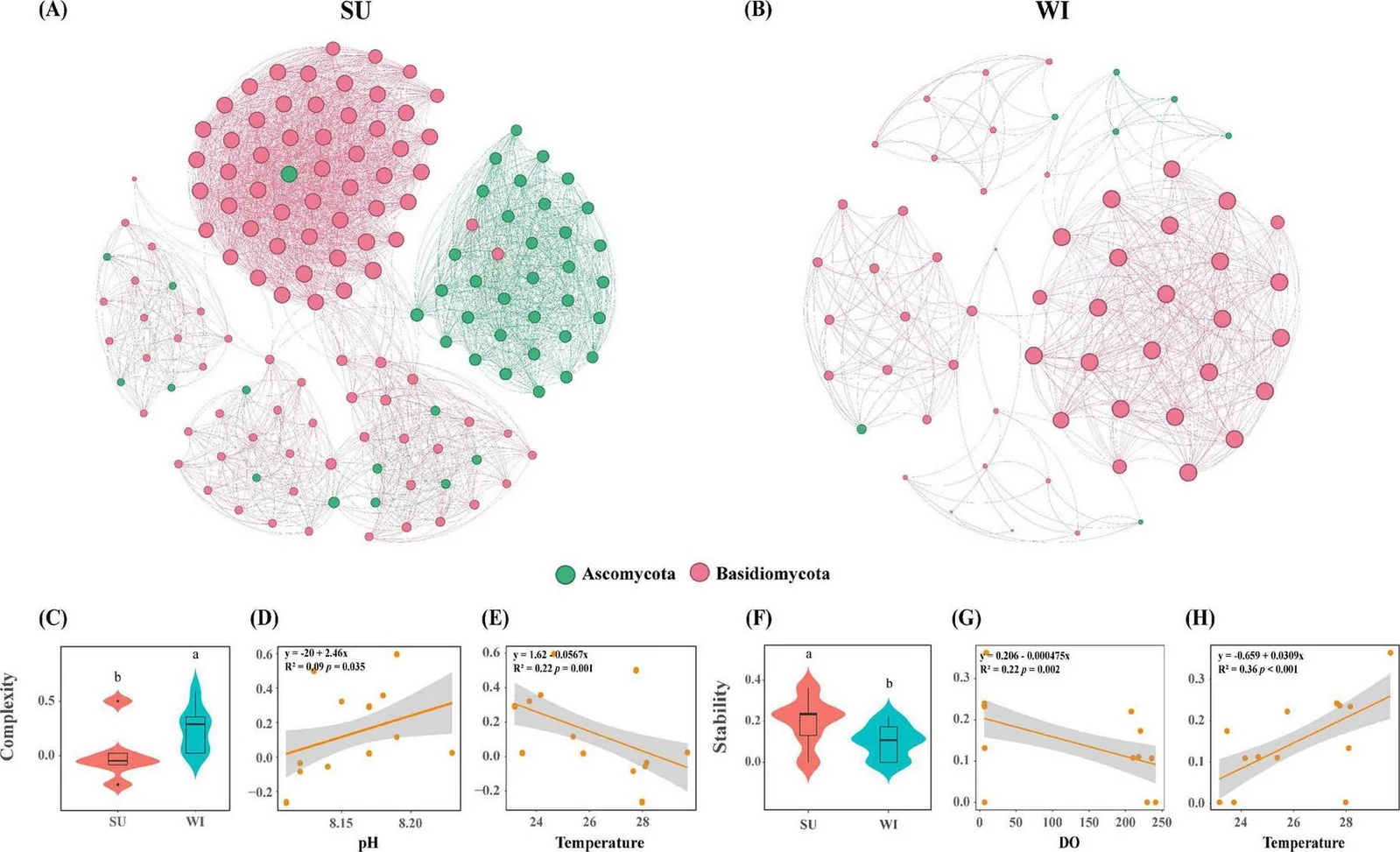
**(A,B)** Co-occurrence network of summer and winter groups of the marine pathogenic fungi. The colors of node represent species belonging to the same module within each network. The pink and green lines represent positive and negative correlations, respectively. **(C)** Complexity in different seasons is presented by a box plot. **(D,E)** Linear regression analyses of complexity against pH and temperature. **(F)** Stability in different seasons is presented by a box plot. **(G,H)** Linear regression analyses of stability against DO and temperature.

Network complexity and stability were represented by cohesion and robustness, respectively. In the marine putative pathogenic bacteria, the summer network had higher complexity (1.605) but lower stability (0.262) than winter (complexity = 1.583, stability = 0.275) (Figure 5). In comparison, the winter network had higher complexity (0.246) than summer (0.015) and lower stability (0.084) than summer (0.108) in the marine putative pathogenic fungi (Figure 6).

### 3.3 Environmental associations with diversity, complexity and stability of the putative marine pathogenic microbial community in the SMS-NWSCS

Spearman correlation analysis and mantel tests were conducted to evaluate the key environmental associations of both alpha and beta diversity of marine putative pathogenic microorganisms. For the putative pathogenic bacterial community, Richness index was significantly influenced by NO_3_^–^-N (r^2^ = −0.62, *p* < 0.001) and NH_4_^+^-N (r^2^ = −0.34, *p* < 0.05) (Supplementary Table 1 and Supplementary Figure 2). For the putative pathogenic fungal community, however, Shannon index was influenced by NH_4_^+^-N (r^2^ = 0.49, *p* < 0.01), while temperature (r^2^ = 0.55, *p* < 0.001) and DO (r^2^ = −0.46, *p* < 0.01) significantly influenced Richness (Supplementary Table 1 and Supplementary Figure 2).

The potential environmental factors that explained the variations in the marine putative pathogenic microbial communities were further assessed by mantel test. In the putative pathogenic bacteria, NH_4_^+^-N (r^2^ = 0.409, *p* < 0.001) emerged as the main factor affecting the marine pathogenic bacterial community in the summer group, while NO_3_^–^-N (r^2^ = 0.545, *p* < 0.001) and temperature (r^2^ = 0.516, *p* < 0.001) were the main factor affecting the winter group. PO_4_^3–^ (r^2^ = 0.545, *p* < 0.001) was the primary factor for the summer group in the putative pathogenic fungal community, while salinity (r^2^ = 0.634, *p* < 0.001), *Chla* (r^2^ = 0.637, *p* < 0.001) and NH_4_^+^-N (r^2^ = 0.637, *p* < 0.001) were the main factors affecting the winter group (Supplementary Table 2 and Figure 7).

**FIGURE 7:**
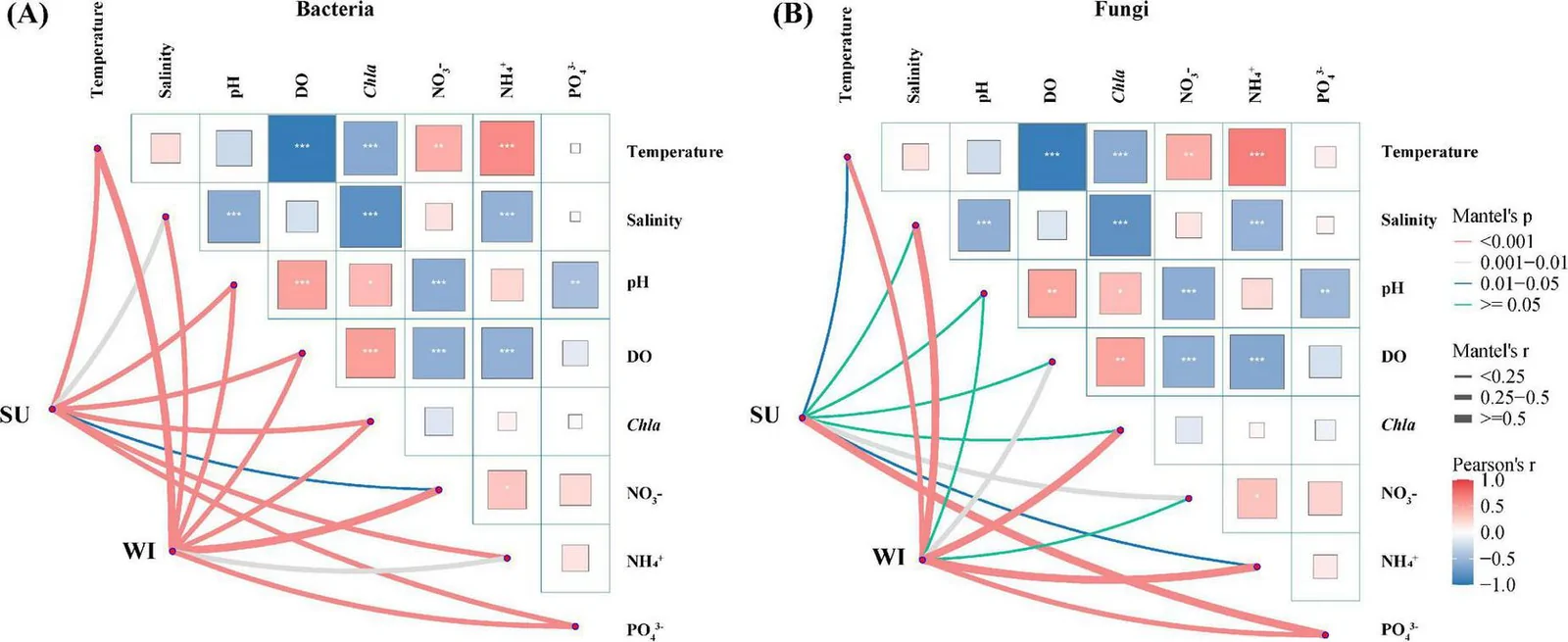
Pair-wise correlations between seasonal groups and environmental factors based on mantel tests. **(A)** Marine pathogenic bacterial community. **(B)** Marine pathogenic fungal community. Line colors represent *P*-values, and the line widths reflect the absolute value of Mantel’s r coefficient.

For the marine putative pathogenic bacteria, the results of spearman’s rank correlation analysis showed that nitrate was positively but pH was negatively correlated with the complexity. Chla and salinity had positive and negative effects on community stability, respectively (Supplementary Table 4). In contrast, pH and temperature had positive and negative effects on marine putative pathogenic fungal community complexity, respectively, whereas DO and temperature had negative and positive effects on community stability of putative pathogenic fungi (Supplementary Table 4).

The relationships among diversity, stability and complexity were evaluate by PLS-PM. In the marine putative pathogenic bacteria, both beta diversity (0.15, *p* < 0.001) and alpha diversity (0.10, *p* < 0.01) showed positive contributions on the community complexity. Moreover, a great negative association with complexity was attributable to water properties (−0.56, *p* < 0.001), while a large positive effect was attributable to nutrients (0.80, *p* < 0.001) (Figures 8A, B, D). For bacterial community stability, alpha diversity (0.45, *p* < 0.001) exerted a positive direct influence, while beta diversity (0.05, *p* > 0.05) showed no association. Water properties (0.38, *p* < 0.001) had a greater positive direct association, while nutrients (−0.16, *p* < 0.01) exhibited a greater negative direct association on stability (Figures 8A, C, D). Both alpha (−0.02, *p* > 0.05) and beta diversity (0.03, *p* > 0.05) showed no association on complexity in the marine putative pathogenic fungi, while the water properties (0.50, *p* < 0.001) and nutrients (0.07, *p* < 0.05) showed a great direct positive association on complexity (Figures 9A, B, D). For the stability of the fungal community, alpha diversity (0.10, *p* < 0.05) had a positive correlation and beta diversity (0.21, *p* < 0.001) exhibited a greater positive association. Additionally, nutrients (0.21, *p* < 0.001) also positively contributed to the stability (Figures 9A, C, D). From the above results, we concluded that compared with beta diversity, alpha diversity had a greater influence on the bacterial community complexity and stability, whereas water properties showed a higher association than nutrients (Figure 8). In the marine putative pathogenic fungal community, beta diversity emerged as the most influential factor for both stability and complexity (Figure 9).

**FIGURE 8:**
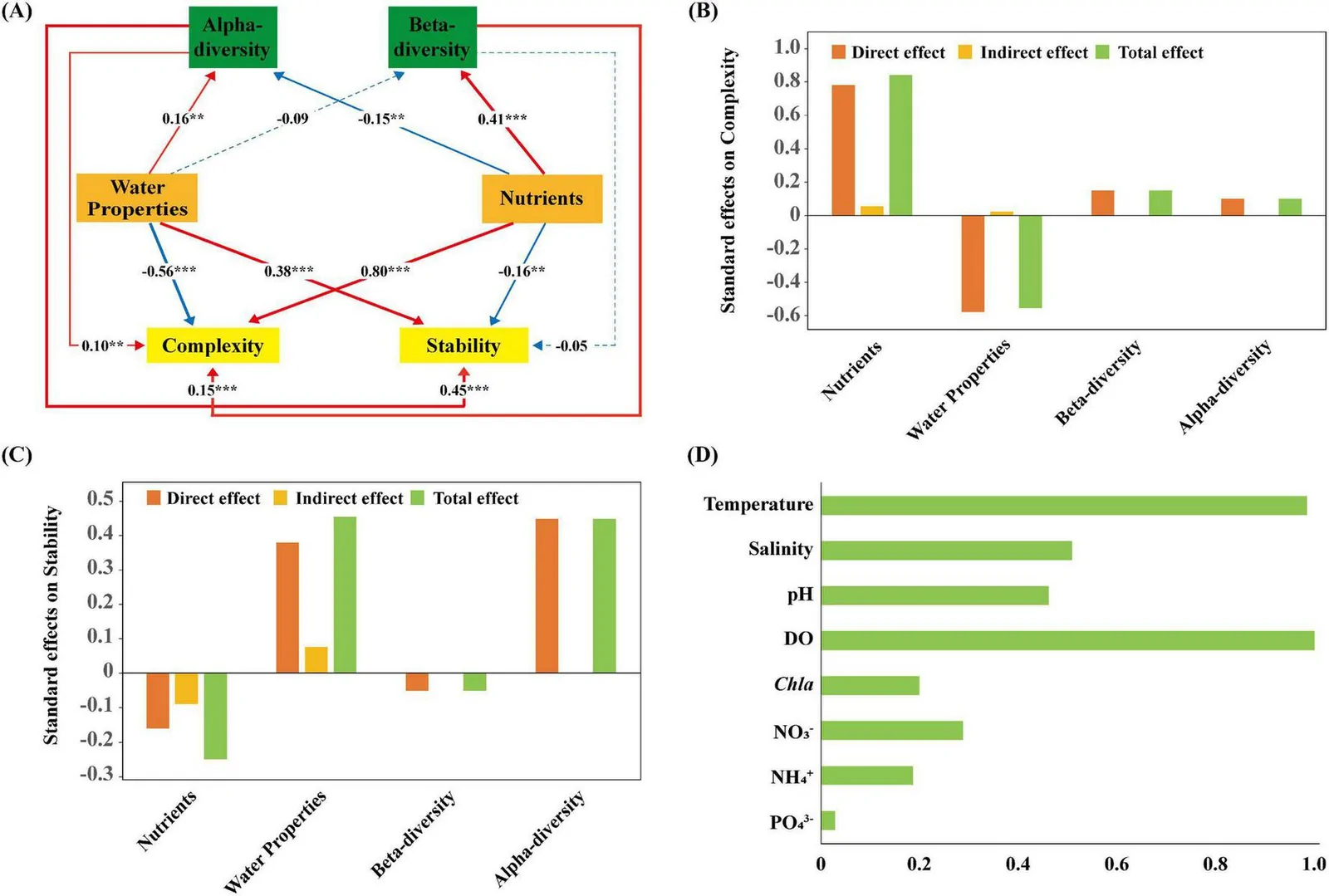
PLS-PM results for the marine bacterial community. **(A)** Red arrows represent positive effects and blue arrows represent negative effects. Solid represent significance and dashed arrows indicate insignificance. The width of solid arrows indicates different significance levels (**p < 0.01; ***p < 0.001), and the numbers represent path coefficients. **(B,C)** Total effects, direct and indirect of different factors on the stability and complexity. **(D)** The individual factor’s contribution to the module. Temperature, salinity, pH and DO, *Chla* are summarized as water properties. NO_3_^–^-N, NH_4_^+^-N, PO_4_^3–^ are summarized as nutrients.

**FIGURE 9:**
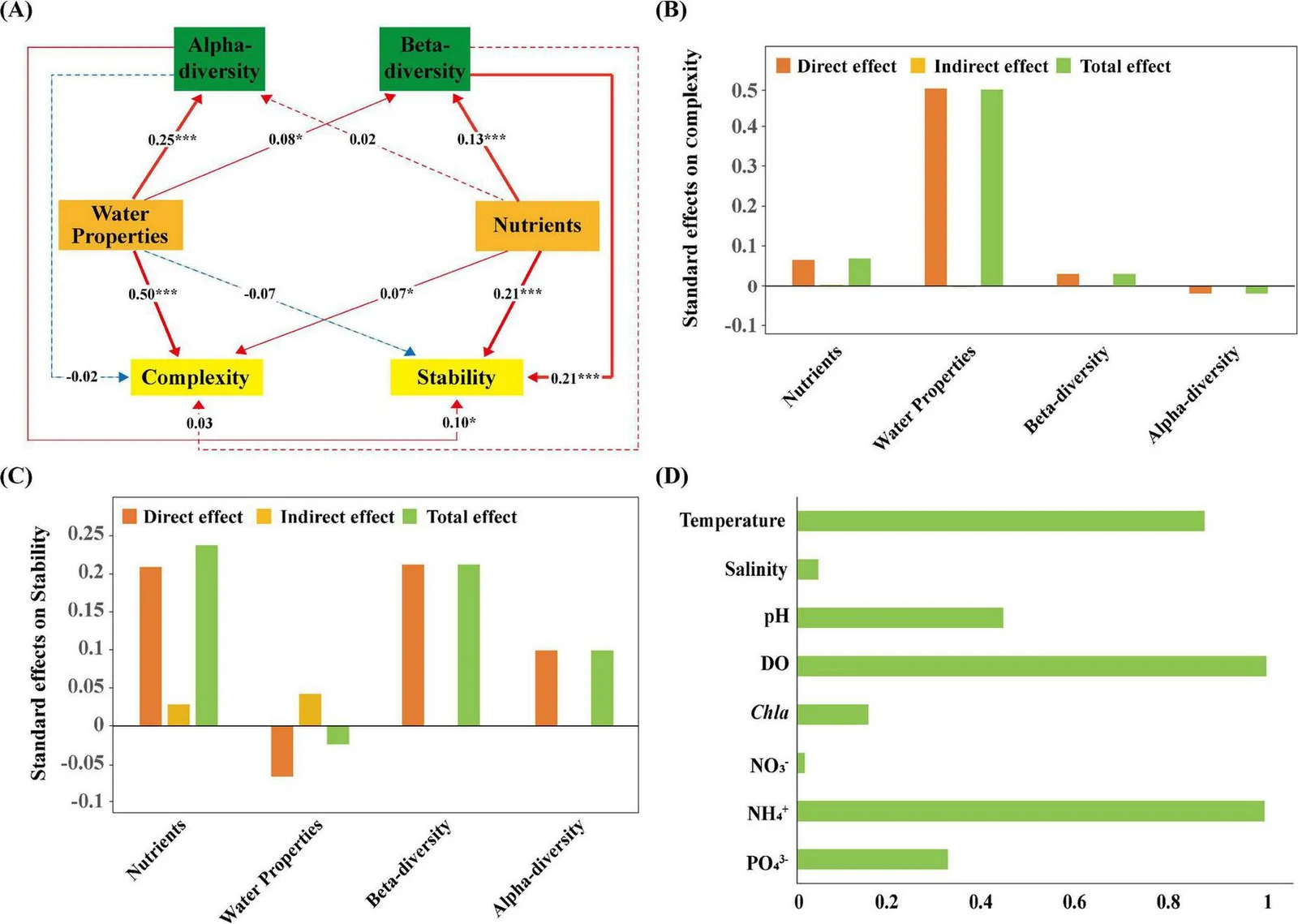
PLS-PM results for the marine fungal community. **(A)** Red arrows represent positive effects and blue arrows represent negative effects. Solid represent significance and dashed arrows indicate insignificance. The width of solid arrows indicates different significance levels (*p < 0.05; ***p < 0.001), and the numbers represent path coefficients. **(B,C)** Total effects, direct and indirect of different factors on the stability and complexity. **(D)** The individual factor’s contribution to the module. Temperature, salinity, pH and DO, *Chla* are summarized as water properties. NO_3_^–^-N, NH_4_^+^-N, PO_4_^3–^ are summarized as nutrients.

## 4 Discussion

### 4.1 *E. coli* and *pseudozyma sp*. dominating putative pathogens in the SMS-NWSCS

Marine pathogens, including bacteria and fungi, affect corals, fish, shellfish and marine mammals, and create significant risks for marine ecosystems, fisheries, aquaculture and public health (Xiang and Wu, 2024). This study provides a systematic survey of the community composition and diversity of marine putative pathogenic microorganisms in the SMS-NWSCS. We identified putative pathogenic bacterial and fungal communities and analyzed their spatial patterns. In the putative pathogenic bacteria, *E. coli*, *P. fluorescens* and *S. paucimobilis* were the dominant species across the SMS-NWSCS. *E. coli*, a well-recognized marine pathogen, infects various shellfish (such as oysters and clams), and has the potential to spread from seafood to humans, causing acute gastroenteritis and other diseases (Vásquez-García et al., 2019). Solo-Gabriele et al. (2000) found *E. coli* was widely distributed in the subtropical coastal environment of Florida. *P. fluorescens* can cause diseases in diverse aquatic species (tilapia, white sea bream etc.), such as rot skin, hemorrhagic, red skin, and ulcer diseases (Guo S. et al., 2022). This bacteria was also common pathogen reported in the subtropical ocean. Previous studies have indicated that *Malassezia* is considered one of the most widespread fungi in the ocean (Steinbach et al., 2023). *Malassezia* can cause dermatitis in marine mammals, such as pinnipeds, and is also associated with coral diseases (Amend, 2014). *Pseudozyma sp.* is also widely distributed in subtropical waters as a fungal pathogen (Gadanho et al., 2003). In this study, we identified *Pseudozyma sp.* and *M. globosa* as the two species with the highest relative abundance. Overall, this study revealed the composition and spatial distribution of dominant putative pathogenic bacteria and fungi in the SMS-NWSCS, and indicated that *E. coli* and *Pseudozyma sp.* may common putative pathogenic microorganisms in the SMS-NWSCS.

In our study, the Shannon index and Richness index of putative pathogenic bacteria both exhibited no significant difference in different seasons (Figure 3A). These results revealed that the species richness and community evenness of putative pathogenic bacteria exhibited relatively stable across seasons. We hypothesize that this seasonal stability may be attributed to the broad ecological niches and strong environmental tolerance of putative pathogenic bacterial species in the community, and that seasonal environmental fluctuations are insufficient to reshape the alpha diversity of the putative pathogenic bacterial community (Grunwald et al., 2022). By contrast, the Shannon index of the putative pathogenic fungal community showed no significant seasonal variation, whereas the Richness index in summer was significantly higher than that in winter (Figure 3B). Consistent with our results, previous studies on mycoplankton in the Yellow Sea have demonstrated that fungal richness and community heterogeneity were higher in summer than in winter and spring, and that temperature was identified as the prime determinant shaping fungal diversity patterns (Wang et al., 2021). The higher summer richness of putative pathogenic fungi may be attributed to warmer summer waters enhanced metabolic activities of marine hosts (plankton, fish, and macroalgae), promoting the release of more organic compounds (polysaccharides, lipids and amino acids) that support more putative pathogenic fungal species (Chrismas et al., 2023). Notably, the increase of new putative pathogenic fungal species in summer likely maintains low relative abundance within the entire community. As a result, the evenness of the putative pathogenic fungal community remains relatively consistent between summer and winter, ultimately leading to no significant difference in the Shannon index across seasons. The results of PCoA analyses showed significant seasonal differences of both marine putative pathogenic bacteria and fungi, and the PERMANOVA further confirmed these results (Figure 4). Guo Y. et al. (2022) demonstrated that putative pathogenic bacteria exhibited strong temporal heterogeneity and were significantly regulated by environmental factors. Our results demonstrated high variability in both putative pathogenic bacterial and fungal communities under environmental heterogeneity. Overall, the SMS-NWSCS environment with gentle seasonal temperature fluctuations can maintain the stability of alpha diversity of putative pathogenic bacterial community, while the enhanced host metabolic activity in summer can enhance the species richness of putative pathogenic fungi, and regulate the beta diversity of both bacterial and fungal communities.

### 4.2 Seasonal difference in network complexity and stability of putative pathogenic bacterial and fungal communities in the SMS-NWSCS

Network cohesion was used to quantify community complexity, reflecting the degree of species association and interaction density within microbial networks (Hernandez et al., 2021; Herren and McMahon, 2017), while network robustness was applied to evaluate community stability, which represents the ability of microbial communities to maintain structural integrity under environmental fluctuation (Zhou et al., 2025). For putative pathogenic bacteria, network cohesion was higher in summer (1.605) than in winter (1.583), indicating a more complex co-occurrence network structure in the summer (Figure 5C). One plausible explanation is that high seawater temperature may weaken host immune defense, potentially promoting cooperative interactions among putative pathogenic bacteria and strengthening interspecific connections (Gnanagobal and Santander, 2022). In contrast, bacterial network robustness was higher in winter (0.275) than in summer (0.262), suggesting that the putative pathogenic bacterial community exhibited greater structural stability and environmental resistance in winter (Figure 5F; Xu et al., 2024). For putative pathogenic fungi, opposite seasonal patterns were observed. Fungal network cohesion was markedly higher in winter (0.246) than in summer (0.015), demonstrating increased network complexity in winter fungal community (Figure 6C), indicating that the fungal community complexity in winter was greater than that in summer (He et al., 2025). A possible explanation is that the reduction of metabolic activity of marine hosts may lower the availability of organic substrates in winter (Girard et al., 2020). Such substrate limitation could intensify competition among putative pathogenic fungi, which may strengthen interspecific associations and contribute to more complex co-occurrence networks. Meanwhile, fungal robustness was higher in summer (0.108) than in winter (0.084), indicating that the putative pathogenic fungal network possessed greater stability during summer periods (Figure 6F). In our study, the species richness of putative pathogenic fungal community was higher in summer. Therefore, we speculate that greater summer richness may support higher functional redundancy, which could increase the resistance of the fungal co-occurrence network (Fang et al., 2025). Overall, the putative pathogenic bacterial community exhibited higher complexity in summer and higher stability in winter, whereas the putative pathogenic fungal community showed higher complexity in winter and higher stability in summer. These divergent seasonal patterns reveal different seasonal adaptation and network assembly strategies between putative pathogenic bacterial and fungal communities in the SMS-NWSCS.

### 4.3 Water properties and nutrients influencing the diversity, complexity and stability of putative pathogenic microbial communities in the SMS-NWSCS

Our analysis revealed that both NO_3_^–^ and NH_4_^+^ were negatively and significantly correlated with the Richness index in marine putative pathogenic bacteria, suggesting that nitrogen availability is a factor associated with alpha diversity (Supplementary Table 1). Consistently, Guo Y. et al. (2022) reported that nutrients, including nitrate, ammonia and phosphate, significantly affect the pathogenic bacterial community. Relatively lower nitrate and ammonium concentrations observed in winter may partially explain the higher bacterial richness detected in winter. In contrast to bacteria, the Shannon index of putative pathogenic fungi was significantly positively correlated with NH_4_^+^ (Supplementary Table 1). As an essential inorganic nitrogen source, ammonium may facilitate fungal growth and metabolic activity, potentially supporting the proliferation of diverse putative pathogenic fungal taxa and further increasing community alpha diversity (Paege et al., 2016). Additionally, fungal richness was positively correlated with temperature and negatively correlated with DO (Supplementary Table 1), which corresponded with the higher fungal richness observed in summer samples. It is speculated that elevated summer temperatures may enhance extracellular enzymatic activity and organic matter decomposition in seawater, which could provide more available substrates for fungal colonization and thereby promote putative pathogenic fungal richness (He et al., 2024). Meanwhile, decreased DO levels driven by summer water stratification and enhanced biological respiration may favor the survival of anaerobic and facultatively anaerobic fungal taxa, potentially contributing to the increased fungal richness in summer (Sato et al., 2017). Mantel test analysis further revealed distinct seasonal environmental factors for the two putative pathogenic communities. For putative pathogenic bacteria, NH_4_^+^ was an important environmental factor in summer, whereas NO_3_^–^ was the important factor in winter (Supplementary Table 2). These may be because microbial decomposition and mineralization are strong in high-temperature water in summer, and a large amount of organic nitrogen was transformed into directly available NH_4_^+^ (Kalinowski et al., 2023), which may result temperature serve as a key limiting resource filtering and structuring summer putative pathogenic bacterial communities. In contrast, high DO conditions in winter may facilitate the nitrification process, promoting the transformation of ammonium to nitrate and rendering nitrate the primary limiting factor for winter bacterial communities (Kalinowski et al., 2023). For putative pathogenic fungi, PO_4_^3–^ was the predominant environmental factors in summer, whereas *Chla* and NH_4_^+^ were the dominant factors in winter. A possible mechanism is that the mycelial growth, lipid metabolism and energy synthesis of putative pathogenic fungi are highly dependent on phosphorus, and PO_4_^3–^ may acts as the main regulatory factor in summer (Tan et al., 2025). The content of *Chla* may also contribute to fungal community dynamics, as phytoplankton represent important substrate and habitat resources for numerous putative pathogenic fungi (Sime-Ngando, 2012). Consequently, fluctuations in phytoplankton biomass could modulate the structure and distribution of putative pathogenic fungal community during winter. The differences of seasonal influential factors observed between putative pathogenic bacteria and fungi reflect distinct nutritional preferences, environmental tolerances, and niche differentiation between the two microbial groups (Čaćković et al., 2023). Overall, our results highlight distinct seasonal patterns and environmental responses between putative pathogenic bacterial and fungal communities in the SMS-NWSCS, implying divergent niche adaptation and regulatory mechanisms underlying their community assembly.

A significant negative association was detected between pH and community complexity, while a positive association was found between nitrate and the complexity of the putative pathogenic bacterial community (Figures 5D, E). We hypothesize that lower pH and higher nitrate may form an environmental stress to putative pathogenic bacterial community, limiting the growth of some putative pathogenic bacteria and potentially enhancing the interaction of the remaining portion in the community (Zhu et al., 2021). For bacterial community stability, salinity and *Chla* were identified as the main influencing factors. We propose that lower salinity and higher *Chla* may reduce salinity stress for the putative pathogenic bacterial community and elevate the supply of organic matter, which could supports richer ecological niches and functional redundancy, potentially enhancing the resistant of putative pathogenic bacterial community (Castellanos et al., 2020). In these results, temperature exhibited significantly negative correlation with complexity and positive correlation with stability of putative pathogenic fungal community (Figures 6E, H). These findings suggest that temperature may act as an important environmental factor regulating the microbial metabolism and community structure of marine putative pathogenic fungi. We inferred that warm summer water may increase the availability of host-derived organic substrates, which could broaden the trophic niche for putative pathogenic fungi and reduce their interspecific competition, thus reducing the complexity and increasing the stability of the community (Zheng et al., 2019). Besides, the high DO and low temperature in winter may reduce the richness of species that are vital to functional redundancy, leading the network more vulnerable despite having higher complexity (Leggieri et al., 2021). These contrasting results of putative bacterial and fungal communities to complexity and stability could be attributed to their fundamental differences in cell components, structure and morphology (Almeida et al., 2023), which determine their tolerance threshold to various environmental factors. Overall, our results demonstrate that factors, including temperature, salinity, pH and *Chla*, are play an important role in regulating the complexity and stability of the putative pathogenic microbial community.

Understanding how community diversity interacts with stability and complexity under environmental perturbations in ecosystems is a vital issue (Xu et al., 2022). PLS-PM results revealed a significant positive association between alpha diversity and both the complexity and stability of the putative pathogenic bacterial community (Figure 8A). Higher alpha diversity generally corresponds to greater species richness and community evenness, which may be linked to elevated functional redundancy and niche complementarity within the putative pathogenic bacterial community (Lupatini et al., 2017). Both alpha and beta diversity exhibited significant positive associations with the stability of the putative pathogenic fungal community (Figure 9A). These findings may suggest that elevated alpha diversity is associated with more efficient resource allocation in the marine putative pathogenic fungal community (Zhou et al., 2025), while higher beta diversity is linked to spatial heterogeneity and functional redundancy in fungal communities (Zhang et al., 2021). In conclusion, this study identified the main environmental factors associated with complexity and stability of marine putative pathogenic microbial communities and provided references for the detection of marine putative pathogenic microorganisms in the SMS-NWSCS.

Although the MBPD and FUNGuild databases are powerful tools for exploring putative pathogenic microorganisms. However, their inherent limitations cannot be overlooked. Amplicon-based annotations relying on these databases cannot distinguish environmental strains and clinically relevant pathogenic strains, nor can they verify actual pathogenicity. Meanwhile, further updates and accumulation of information on marine pathogenic species will help improve their predictive capacity for researchers (Wu et al., 2023).

## 5 Conclusion

This study elucidated the seasonal patterns in diversity, complexity and stability of marine putative pathogenic microbial communities in the subtropical SMS-NWSCS. It was found that *E. coli*, *P. fluorescens* and *S. paucimobilis* were the dominant putative pathogenic bacteria, while *Pseudozyma sp.* and *M. globosa* were the dominant putative pathogenic fungi. The alpha diversity of putative pathogenic bacteria was higher in winter, while fungal alpha diversity was higher in summer. Beta diversity exhibited significant seasonal dynamics for both putative pathogenic bacterial and fungal communities. Spearman’s correlation analysis revealed that a suite of environmental factors, including temperature, pH, DO, *Chla*, nitrate, ammonium and phosphate, were associated with the variation of putative pathogenic microbial community structure. PLS-PM analysis demonstrated that bacterial alpha diversity was positively associated with community complexity and stability, and both alpha and beta diversity showed positive correlations with the stability of the putative pathogenic fungal community. Overall, this study offers a novel perspective on marine pathogenic microorganisms, enhancing our understanding of the maintenance mechanisms and ecological factors associated with pathogenic microbial communities in the SMS-NWSCS.

## Data Availability

The data analyzed in this study are available in the NCBI repository (https://www.ncbi.nlm.nih.gov) under BioProject accession numbers PRJNA1469362 and PRJNA1469374.
