# Supplementary material for: Seasonal community structure and environmental associations of putative pathogenic microorganisms in the Shelf-Margin Sea of the Northwestern South China Sea
Source: Front Microbiol. 2026 Oct 26;17:1980205. doi: 10.3389/fmicb.2026.1980205 (PMC13745369; doi:10.3389/fmicb.2026.1980205)
Supplement: Supplementary file 1 [file Table_1.docx]

**Supplementary**

Figure 1 Relative abundance of marine putative pathogenic microorganisms in different sites. (A) marine putative pathogenic bacteria. (B) marine putative pathogenic fungi.

Figure 2 Linear regressions for alpha diversity of the marine putative pathogenic microbial community. (A-C) Marine putative pathogenic bacterial community. (D-F) Marine putative pathogenic fungal community.

Table 1 Spearman tests for the correlations between alpha diversity index and the environmental factors.

|  | Bacteria | | Fungi | |
| --- | --- | --- | --- | --- |
|  | Shannon | Richness | Shannon | Richness |
| Temperature | -0.28 | -0.14 | 0.13 | 0.55*** |
| Salinity | -0.07 | 0 | -0.25 | 0.34* |
| pH | -0.05 | 0.29 | 0.17 | -0.03 |
| DO | -0.1 | 0.15 | -0.01 | -0.46** |
| *Chla* | 0.21 | -0.09 | 0.35* | -0.2 |
| NO_3_^-^ | -0.2 | -0.62*** | -0.01 | 0.15 |
| NH_4_^+^ | 0.25 | -0.34* | 0.49** | 0.33* |
| PO_4_^3-^ | 0.3 | 0.17 | 0.13 | -0.4* |

Table 2 Mantel tests for the correlations between beta diversity and the distance of individual environmental factors.

|  | Bacteria | | Fungi | |
| --- | --- | --- | --- | --- |
|  | SU | WI | SU | WI |
| Temperature | 0.300*** | 0.516*** | 0.156* | 0.369*** |
| Salinity | 0.316** | 0.336*** | 0.109 | 0.634*** |
| pH | 0.323** | 0.424*** | 0.118 | 0.035 |
| DO | 0.289*** | 0.430*** | 0.08 | 0.316*** |
| *Chla* | 0.280*** | 0.373*** | 0.087 | 0.637*** |
| NO_3_^-^ | 0.200* | 0.545*** | 0.251** | 0.119 |
| NH_4_^+^ | 0.409*** | 0.306** | 0.238** | 0.637*** |
| PO_4_^3-^ | 0.384*** | 0.498*** | 0.545*** | 0.374*** |

SU: Summer; WI: Winter

Table 3 Topological parameters of the marine putative pathogenic microbial co-occurrence networks for the two groups.

|  | Bacteria | | Fungi | |
| --- | --- | --- | --- | --- |
|  | SU | WI | SU | WI |
| Nodes | 100 | 140 | 146 | 60 |
| Edges | 534 | 1049 | 2510 | 432 |
| Average degree | 10.68 | 14.99 | 34.38 | 14.40 |
| Diameter | 5 | 5 | 5 | 7 |
| Density | 0.108 | 0.108 | 0.237 | 0.244 |
| Clustering coefficient | 0.512 | 0.479 | 0.944 | 0.932 |
| Modules | 7 | 7 | 5 | 6 |
| Modularity | 0.555 | 0.456 | 0.614 | 0.496 |
| Positive edges | 303 | 624 | 2501 | 418 |
| Negative edges | 231 | 425 | 9 | 14 |
| Complexity | 1.605 | 1.583 | 0.015 | 0.246 |
| Stability | 0.262 | 0.275 | 0.108 | 0.084 |

SU: Summer; WI: Winter

Table 4 Spearman tests for the correlations among complexity, stability, and environmental factors in the marine putative pathogenic microorganisms.

|  | Bacteria | | Fungi | |
| --- | --- | --- | --- | --- |
|  | Complexity | Stability | Complexity | Stability |
| Temperature | 0.52*** | -0.34* | -0.48** | 0.59*** |
| Salinity | 0.03 | -0.52*** | -0.12 | 0.29 |
| pH | -0.53*** | 0.25 | 0.58*** | -0.01 |
| DO | -0.34* | 0.36* | 0.47** | -0.65*** |
| *Chla* | -0.35* | 0.48** | 0.4* | -0.32* |
| NO_3_^-^ | 0.68*** | -0.05 | -0.37* | -0.13 |
| NH_4_^+^ | 0.26 | 0.1 | -0.32* | 0.3 |
| PO_4_^3-^ | -0.18 | -0.11 | 0.09 | -0.17 |
